# Stakeholder Perspectives on the Responsible Innovation in Health Framework for Addressing Stigma‐Based Health Inequalities Among People Who Stutter

**DOI:** 10.1111/1460-6984.70115

**Published:** 2025-08-21

**Authors:** Sébastien Finlay, Geneviève Lamoureux, Anne Moïse‐Richard, Lucie Ménard, Ingrid Verduyckt

**Affiliations:** ^1^ School of Speech‐Language Pathology and Audiology Université de Montréal Montreal Quebec Canada; ^2^ Centre for Interdisciplinary Research in Rehabilitation of Greater Montreal Montreal Quebec Canada; ^3^ Marie Enfant Rehabilitation Centre of Sainte‐Justine University Hospital Centre Montreal Quebec Canada; ^4^ Laboratoire de Phonétique, Département de linguistique Université du Québec à Montréal Montreal Quebec Canada

**Keywords:** concept mapping, health inequalities, Responsible Innovation in Health, stigma, stuttering

## Abstract

**Objectives:**

People who stutter (PWS) experience stigma‐based health inequalities that can negatively impact their quality of life. Yet, few interventions in the literature are explicitly designed to address these systemic disparities. The Responsible Innovation in Health (RIH) framework offers a promising foundation for developing health innovations that are equitable, sustainable and contextually responsive. This study explores how stakeholders interpret and apply the RIH framework to envision interventions that reduce stigma and promote health equity for PWS.

**Methods:**

Using a mixed‐methods design, namely the Participative Concept Mapping Approach, stakeholders (PWS, clinicians, health innovators) participated in a workshop to generate, sort and rate ideas based on their importance and feasibility. Concept maps were used to analyse and categorize ideas thematically.

**Results:**

Stakeholders generated 94 ideas across six clusters as follows: (1) Digital Technology and Video Media, (2) Collective and Professional Approaches, (3) Cost and Accessibility, (4) Inclusive and Sustainable Intervention Design, (5) Engaging Multi‐Modal Approaches and (6) Flexibility. The environmental responsibility value of the RIH framework received limited focus. Discrepancies between the importance and feasibility of ideas highlighted challenges in implementing interventions, while ensuring their sustainability.

**Conclusion:**

This study demonstrates how stakeholders prioritize values of the RIH framework when envisioning stigma‐reducing health innovations for PWS. Findings highlight the need for embedding sustainability within clinical practices and underscore the importance of patient and user feedback to bridge the gap between impactful concepts and practical solutions, ensuring that interventions are meaningful, feasible and grounded in the RIH values.

**WHAT THIS PAPER ADDS:**

*What is already known on this subject*

- Stuttering is associated with stigma‐based health inequalities that extend beyond speech, impacting PWS across multiple domains of life. Research has documented the effects of stigma on quality of life, access to care and communicative participation for PWS. However, frameworks to guide the development of interventions that explicitly address these structural inequities remain limited.

*What this paper adds to existing knowledge*

- This study is the first to explore how the RIH framework can inform the design of stigma‐reducing interventions tailored to stuttering. Through participatory concept mapping with PWS, clinicians and health innovators, it identifies how RIH principles, such as inclusion, sustainability and responsiveness, can shape context‐specific, equity‐oriented innovations that reflect the lived experiences of PWS.

*What are the potential or actual clinical implications of this work?*

- Applying the RIH framework to stuttering interventions offers a novel approach for promoting health equity in both clinical and community contexts. This study highlights the importance of co‐designing interventions with stakeholders, grounding them in the lived experiences and priorities of people who stutter. It sets the stage for developing sustainable, meaningful and inclusive practices that respond to the complex realities of stigma in stuttering.

## 1 Introduction

Traditionally defined as a fluency disorder, stuttering has long been conceptualized as an individual deficit to be corrected (Lamoureux, Tessier, et al. 2024; St. Pierre and St. Pierre 2018). However, this reductionist view fails to account for the broader realities faced by people who stutter (PWS) (St. Pierre 2013). For PWS, stuttering is experienced as a range of behavioural, emotional and cognitive reactions that go beyond the moments of speech that may be perceived as disfluent by listeners (Tichenor and Yaruss 2019). These experiences are not just private or clinical, they are socially shaped and politically charged (Constantino et al. 2022). Such experiences can have lasting effects on identity, communication and participation in daily life (Gerlach et al. 2021).

Recent research highlights that stuttering is shaped not only by internal responses but also by the social environments in which communication occurs (Boyle 2017; Constantino 2018; Constantino et al. 2020). Negative social responses can heighten the emotional toll of stuttering and reinforce patterns of avoidance or concealment (Bricker‐Katz et al. 2009). In institutional contexts, such as healthcare or education, communication norms that favour speed and fluency can lead to misunderstandings, exclusion or unequal access to services (Adriaensens et al. 2017; Perez et al. 2015). These insights resonate with the social model of disability, which reframes disability not as a deficit located within the individual, but as a consequence of environmental, attitudinal and structural barriers (Constantino et al. 2022; Isaacs and Swartz 2022). From this perspective, it is not stuttering itself but society's response—rooted in ableist norms about speech—that disables individuals (St. Pierre 2017; Shenker et al. 2023). Accordingly, stuttering can be understood as a complex health and social phenomenon that intersects with broader issues of stigma, marginalization and health justice (Boyle et al. 2023; Gerlach et al. 2021; Gerlach‐Houck et al. 2018).

Building on this understanding, this article explores how the Responsible Innovation in Health (RIH) framework can help guide the design of interventions that are socially inclusive and responsive to the stigma‐based health inequities experienced by PWS. Throughout the article, the terms innovation and intervention are used interchangeably to refer to programs, actions or practices aimed at supporting PWS and improving their experiences, access to care and health and social outcomes.

### 1.1 Stigma‐Based Health Inequalities and Stuttering

Health inequalities are defined as ‘the systematic, avoidable and unfair differences in health outcomes that can be observed between populations, between social groups within the same population or as a gradient across a population ranked by social position’ (McCartney et al. 2019). Stigma—a known cause of such inequalities—disproportionately affects vulnerable groups, including individuals with communication differences such as stuttering (Hatzenbuehler et al. 2013). Stigma‐based barriers often restrict access to fundamental social determinants of health, such as employment, housing and education, which further widen health disparities (Link and Hatzenbuehler 2016). Moreover, stigma acts as a barrier to treatment engagement and adherence, further exacerbating health inequalities (Arnaez et al. 2020).

Specifically, PWS are at risk of experiencing reduced quality of life across their lifespan due to stigma‐based health inequalities (Boyle and Cheyne 2024; Boyle et al. 2023). These inequalities emerge not from stuttering itself, but from the social responses and structural barriers that PWS encounter in their environments. For example, PWS often report negative healthcare experiences, including providers’ lack of understanding, time constraints and communication challenges (Boyle and Fearon 2018; Bricker‐Katz et al. 2010). Such experiences can discourage help‐seeking and result in unmet healthcare needs. A growing body of research shows that internalized stigma—comprising anticipated stigma, felt stigma and self‐applied negative beliefs—significantly predicts poorer mental health and reduced communicative participation (Boyle et al. 2023; Gerlach‐Houck et al. 2021). This internalization is shaped by early experiences of social devaluation and by societal norms that pathologize non‐normative speech (Tichenor et al. 2022).

To manage this stigma, many PWS develop concealment strategies to hide their identity as a PWS due to stigma, including avoiding certain words and situations, altering their speech patterns (e.g., substituting words) and remaining silent (Connery et al. 2020). Although intended to shield against anticipated judgment, they are consistently associated with increased psychological distress, reduced communicative participation and feelings of inauthenticity, and are linked to a diminished quality of life and heightened psychological distress in PWS (Boyle et al. 2018; Gerlach‐Houck et al. 2023; Gerlach‐Houck et al. 2021). They have been linked to diminished quality of life and mirror coping mechanisms observed in other marginalized groups experiencing stigma‐related health disparities. These patterns underscore the importance of viewing stuttering through a health equity lens and call for systemic responses that go beyond individual adaptation.

While the impact of stigma on health outcomes is well documented, few frameworks exist to guide the design of interventions that actively respond to these systemic inequities. This omission is particularly visible in fields like speech‐language pathology, where interventions are often designed around normalization goals (Constantino et al. 2022; St. Pierre 2013), with limited attention to equity, inclusion or the broader social conditions that shape access and outcomes. This gap is particularly visible in communication‐related interventions, including those for stuttering. The RIH framework offers a promising alternative by supporting the design of interventions that align with public values and respond to population‐level health needs.

### 1.2 From RRI to RIH: Conceptual Foundations for Responsible Health Innovation

One framework that seeks to operationalize these broader commitments to inclusion and justice is RIH, a health sector–specific adaptation of the Responsible Research and Innovation (RRI) model. The RRI framework, defined as a ‘design strategy which drives innovation and gives some “steer” towards achieving societal desirable goals’ (Von Schomberg 2013), provides a structured approach to navigating these changes. With its foundation in four core dimensions—inclusion, anticipation, responsiveness and reflexivity—RRI offers a proactive approach to align innovation processes with public values, aiming to integrate a broad range of societal voices and address complex societal challenges from an early stage. Over time, additional dimensions such as sustainability and care have emerged in RRI to address the importance of long‐term viability and ethical responsibility, particularly within health systems where both social justice and sustainability are critical (Burget et al. 2017). However, the general framework of RRI has faced critiques regarding its applicability in specific sectors like healthcare, where more tailored guidelines are needed to navigate the unique challenges of health systems and the ethical complexities of health inequalities (Silva et al. 2018).

In response to this criticism, Silva et al. (2018) introduced the RIH framework, which builds on RRI principles to address health system needs and align innovation with public health values. The RIH framework, developed through both deductive and inductive theory‐building approaches, defines nine dimensions across five value domains—population health, health systems, economic value, organizational value and environmental value—aimed at ensuring the relevance, equity and sustainability of health innovations throughout their lifecycle. By integrating insights from health technology assessment, health systems literature and entrepreneurship, RIH emphasizes innovations that not only address immediate clinical needs but also contribute to systemic health equity and sustainability (Silva et al. 2018). Furthermore, the RIH framework offers specific guidance on how health innovations can be both ethically developed and contextually responsive, highlighting the need for solutions that respect the social, economic and environmental dimensions of healthcare. This tailored framework thus operationalizes the RRI principles in ways that directly address health sector‐specific issues, particularly by aligning innovation development with population health and health system needs (Silva et al. 2021).

### 1.3 Current Landscape of Interventions for Stuttering in Canada

Our study focuses on stuttering in the province of Québec, Canada, where access for PWS to speech‐language therapy services presents challenges. Public services are primarily available to minors and often involve waitlists exceeding 1 year. In this context, PWS have limited access to health promotion campaigns or services specifically tailored to their needs, further compounding the challenges they face in accessing adequate care. This context underscores the importance of locally relevant, practical interventions grounded in the RIH framework, ensuring that health innovations are both responsive and sustainable within Québec's health landscape.

### 1.4 The Present Study

Despite the relevance of the RIH framework for addressing stigma‐based health inequalities, little is known about how it can be applied to design interventions for communication differences such as stuttering. To date, no studies have examined how stakeholders collectively interpret and prioritize the values embedded in this framework. Understanding these perspectives is essential for ensuring that future interventions are not only clinically relevant but also socially inclusive and structurally responsive. This study aimed to understand how key stakeholders, including clinicians, people with lived experience of stuttering, and health innovators (HIs), interpret and apply the RIH framework to envision a stigma‐reducing intervention for stuttering. During a 3‐h workshop, we introduced the RIH framework to participants, encouraging them to consider how its principles might guide the creation of stigma‐reducing interventions. Through participatory concept mapping, stakeholders then generated, sorted and rated ideas based on their perceived importance and feasibility. This process provided insights into how different groups prioritize values within the RIH framework.

## 2 Materials and Methods

### 2.1 Design

This study employed a mixed‐methods design based on the Participative Concept Mapping Approach (PCMA), integrating a structured participatory process with statistical analyses, such as multi‐dimensional scaling and hierarchical cluster analysis. This approach combined quantitative and qualitative data to foster collaboration and gather diverse insights. Concept mapping is known to effectively integrate multiple knowledge sources to address complex public health challenges (van Bon‐Martens et al. 2017). Unlike traditional qualitative methods that rely on researcher‐led interpretation, the PCMA is driven by stakeholder input, with thematic structures emerging directly from how participants sort ideas. The Research Ethics Committee in Education and Psychology at Université de Montréal waived the requirement for an ethics review, classifying the study as expert consultation. Participants were consultants, in line with Canada's Tri‐Council Policy Statement.

### 2.2 Participants and Recruitment

The participants reported on in this paper were part of a bigger project on the use of concept mapping and the stigma of stuttering (Lamoureux et al. 2024). Participants were invited to workshops, and this paper focuses on a specific workshop where stakeholders interpreted and applied the RIH framework to envision an intervention to alleviate stigma‐based health inequalities of stuttering. Participants for the study were purposively recruited. The inclusion criteria included participants (1) from three stakeholder groups (i.e., HIs, clinician and PWS), (2) who were 18 years and older and (3) who had a comprehensive understanding of the French language. HIs were defined as individuals engaged in developing, evaluating or implementing health interventions or technologies, including professionals working in digital health, healthcare design or innovation strategy, with or without direct experience in communication‐related disabilities. Recruitment relied on professional and community networks. PWS were contacted via advocacy organizations and local community groups, such as the Association Bégaiement Communication; clinicians were recruited through provincial professional networks and mailing lists, such as the Québec Community of Practice in Stuttering and Cluttering and HIs were approached based on their prior participation in responsible innovation workshops, involvement in patient‐partnered design projects or affiliation with innovation hubs in Québec. All participants self‐identified as either having lived experience with stuttering (*n* = 5), working clinically with PWS (*n* = 6) or contributing to health innovation (*n* = 6).

Demographic data are summarized as follows. Among the PWS (mean age = 40.6 years; range = 25–65), there were three women and two men, primarily identifying as Caucasian (*n* = 4) or Afro‐descendant (*n* = 1), all residing in Québec, Canada. Educational backgrounds included one diploma of vocational studies, one college diploma and three bachelor's degrees. The clinicians (mean age = 40.33 years; range = 28–47) included five women and one man, all self‐identified as Caucasian. They were based in Québec (*n* = 4) and France (*n* = 2) and held five master's degrees and one doctorate. This group included four speech‐language therapists (SLTs), one social worker and one psychologist. The HIs (mean age = 40.33 years; range = 31–53) included five women and one man, with ethnic backgrounds reported as Caucasian (*n* = 4), Arabic (*n* = 1) and mixed ethnicity (*n* = 1). All were based in Québec and held advanced degrees (one master's, five doctorates), with areas of expertise in health innovation, disability and stigma through arts and technology (*n* = 5), and the sociology and politics of disability, inclusion and diversity (*n* = 1).

### 2.3 Data Generation

The PCMA process was followed as outlined by Kane and Trochim (2007), encompassing six steps: (1) preparation; (2) generation of ideas; (3) structuring of ideas via rating and sorting; (4) data analysis and representation of ideas; (5) interpretation of results and (6) application of outcomes and maps. A detailed description of each step is provided in Lamoureux et al. (2024). Based on this framework, we developed a detailed facilitation guide to ensure methodological consistency across workshops and alignment with best practices in participatory concept mapping. Data was collected during two workshops conducted via Zoom in French. The workshops were facilitated by two members of the research team (S.F. and G.L.), both of whom are PWS, SLTs and researchers in the field of speech‐language pathology. The first workshop, lasting 3 h, was organized as follows: the first hour was dedicated to an introduction to the RIH framework (Silva et al. 2018), with an emphasis on examining its values as both synergistic and potentially conflicting, illustrating the inherent complexity of applying the framework. Following this introduction, participants engaged in discussions to reflect on how the values within the RIH framework could be applied to various health innovations. During the second hour, a structured brainstorming session was conducted, guided by the prompt: ‘In light of the principles of responsible innovation in health, when envisioning an intervention addressing (self‐)stigma in stuttering, it should…’. This prompt was pilot tested prior to the workshop to ensure clarity and relevance. Notably, the term ‘(self‐)stigma’ was chosen to highlight stigma's varying levels, encompassing both self‐stigma and societal stigma. During the idea generation process, all stakeholders could view each other's ideas in real‐time. The research team, while discussing with stakeholders, made on‐the‐spot modifications, such as merging ideas that were deemed too similar. These merging decisions were made in real time by mutual agreement between participants. As stakeholders could see the idea list update live on screen, any proposed merges by the research team were explicitly discussed and only applied when participants agreed the content overlapped significantly. In the third hour, participants were invited to sort all ideas thematically into clusters using an inductive approach. Each participant independently grouped ideas based on perceived conceptual similarity and assigned a label to each cluster, reflecting their interpretation of the underlying theme. Participants also rated each idea based on two indicators, namely importance and feasibility, selected by the research team as relevant for the context. The second workshop, which lasted two hours, was dedicated to interpreting and validating the concept maps created from the data. The research team presented the results from the workshop, followed by a discussion with the stakeholders to ensure that their perspectives and insights were included to contribute to the validity and relevance of the findings.

### 2.4 Data Analysis

The data were analysed using RCmap, an open‐source software for concept mapping (Bar and Mentch 2017) and IBM SPSS Statistics 29. Two types of concept maps were created and examined in this study. The first map, the Cluster Map, was generated using RCmap and displays stakeholder‐generated ideas on a two‐dimensional graph where points are grouped into clusters represented by convex polygons. In this map, the proximity of points indicates the frequency with which ideas were sorted into the same cluster by participants. RCmap initially suggests potential cluster configurations through multi‐dimensional scaling, based on the data. This tool supports the research team in organizing ideas that reflect common perceptions within the group rather than making definitive decisions on grouping. During the second workshop, the research team presented the preliminary Cluster Map for group discussion. Stakeholders were encouraged to voice concerns about idea placements and propose adjustments. Reassignment stemmed from participant‐led reflections on thematic fit. All changes were made through open discussion until verbal consensus was reached, with the research team acting solely as facilitators to document the agreed‐upon changes. For this study, several cluster solutions were explored (ranging from two to twelve clusters), and the six‐cluster configuration was selected as the optimal balance between statistical coherence and conceptual clarity. This solution provided distinct thematic groupings without over‐fragmenting related ideas. Specifically, this configuration yielded a within‐cluster sum of squares value of 9, indicating relatively low dispersion within clusters (with lower values reflecting tighter groupings). It also produced an average silhouette value of 2, which refers to the average number of clusters into which each idea was sorted by participants. With this configuration, values range from 1 (perfect agreement) to a maximum of 6 (the total number of clusters), with lower values indicating greater consistency in how participants conceptualized the ideas. This configuration resulted in a misplacement index of 0.25, which is generally considered acceptable in concept mapping analyses as it suggests that 75% of the points are consistent with participants’ original sorting. While there is no universal cut‐off, values below 0.30 are commonly interpreted as indicating good fit between participant data and the cluster solution (Kane and Trochim 2007). The second map, the Go‐Zone Graph, was created using IBM SPSS Statistics 29 and visualizes stakeholder ratings based on importance and feasibility. Each idea is represented as a point, allowing simultaneous assessment of its importance (X‐axis) and feasibility (Y‐axis). Points closer to the origin are rated as less important or less feasible. Vertical and horizontal lines denote the average values for importance and feasibility, dividing the graph into four quadrants for easier interpretation: Quadrant 1 (low importance, low feasibility), Quadrant 2 (high importance, low feasibility), Quadrant 3 (low importance, high feasibility), and Quadrant 4 (high importance, high feasibility). This quadrant system offers a structured way to prioritize ideas but still serving as a flexible guide rather than a strict directive.

## 3 Results

### 3.1 Identified Ideas

The comprehensive details of the 94 generated ideas are presented in Table 1, including mean values for importance and feasibility ratings, as well as the quadrant positioning in relation to the Go‐Zone graph. As explained in the methods section, some ideas were merged during the generation process, which explains why certain ideas are identified with numbers exceeding 94.

**TABLE 1:** Ratings of all stakeholders' generated ideas sorted according to their clusters.

		Mean rating (1–5)		
		Importance	Feasibility	Go‐Zone quadrant
Cluster 1: Digital Technology and Video Media		3.86	3.27	
1	Propose a free application linked to speech therapy monitoring (with the possibility of discussing/sharing within a community, having reminders for set challenges…)	3.94	3.24	2
10	Include videos or an educational application.	3.94	3.65	4
15	Suggest the use of virtual reality software to gradually expose oneself to speaking situations.	3.88	3.00	2
36	Ensure confidential data handling (secure platform).	4.94	4.24	4
42	Use virtual reality to allow a person who doesn't stutter to interact 'in the shoes' of a person who stutter.	3.65	3.00	1
47	Develop a video game for people who stutter.	3.35	2.71	1
51	If technology‐based, require minimal updates.	4.12	3.06	2
59	Utilize virtual reality to help practice reacting to stigmatization.	3.82	3.12	2
61	Virtual approach for the close family or friends of the person who stutters.	3.76	3.53	1
64	Sims‐style role‐playing games where people who stutter envision their desired outcomes and how to achieve them.	3.06	2.71	1
74	Engage various channels (films, audio, virtual reality).	3.88	3.35	2
76	Use social media to create a sort of community among groups (Facebook group, Slack, WhatsApp, Discord, etc.).	3.76	3.94	3
77	Creating a virtual video game, a kind of mission or story where you are the hero (PWS), could involve filming the story and using it as a basis for awareness‐raising films for the public.	3.29	2.47	1
110	Maintain a strong ethical consideration around the use of artificial intelligence if employed.	4.59	3.76	4
Cluster 2: Collaboration, Experiential Learning and Deliverables for a Comprehensive Approach to (Self‐)stigma Intervention		4.18	3.98	
13	Facilitate collaboration among different participants (general population, individuals who stutter, caregivers) around a simple project.	4.06	3.41	2
27	Propose therapeutic groups for both patients and parents, conducted in‐person and online.	4.35	4.00	4
28	Promote group cohesion before delving into the main content (e.g., a quick group activity before delving into the main topic).	3.94	4.24	4
33	Include group sessions with other people who stutter.	4.24	4.12	4
34	Allow for experiential learning.	4.65	4.12	4
38	Replicate everyday life situations.	4.65	3.94	4
39	Maintain a professional approach.	4.71	4.65	4
40	Enable progress tracking.	4.18	3.71	4
66	Request clients who stutter to actively contribute their experiences (due to the wide variety in the experiences of people who stutter).	4.65	4.53	4
70	Review the chosen approach with the patient after a few weeks.	4.53	4.18	4
90	Incorporate examples of stuttering (audio or video excerpts).	3.94	4.29	4
97	Include practical exercises to identify and react to stigma and self‐stigma.	4.24	3.71	4
99	For new approaches: Let people who stutter provide their input and behaviour for testing.	4.41	3.94	4
104	Include homework assignments.	4.12	4.00	4
107	Examine the patient's personal history to comprehend the roots of self‐stigmatization. A psychological approach is fundamental to therapy design.	3.88	3.29	2
2	Incorporate a theoretical section for a comprehensive understanding of stigma and self‐stigma.	4.18	4.29	4
3	Provide a summary pamphlet at the end of the intervention as a ‘deliverable’ for participants to keep and refer back to later.	3.76	4.29	3
69	Develop an Intervention Kit (so anyone knowledgeable in the subject can organize the intervention locally).	3.59	3.06	1
109	Document relevant data (e.g., information the people who stutter would like to track/retain).	4.41	4.06	4
62	Offer recognition to participants (sensitization card, badge, small certificate).	3.18	4.41	3
72	Utilize artistic co‐creation to raise awareness among the public (emotion = memory) and foster a supportive and empowering atmosphere for PWS.	4.18	3.29	2
Cluster 3: Cost and Accessibility		4.28	3.27	
46	Ensure intervention is free (funded by the city, government, association, related activity).	4.35	2.35	2
23	Vary costs based on individual income.	3.47	2.59	1
81	Secure government funding.	4.71	2.41	2
82	Obtain funding from a non‐profit organization (NPO).	3.71	2.59	1
83	Fund through public funds.	4.53	2.41	2
87	Involve students (offer internships).	3.47	3.35	1
92	Lower costs by partnering with major companies (increase accessibility).	3.29	2.18	1
100	Seek funding from foundations, associations, state, regional authorities or social security.	4.29	2.59	2
4	Prioritize affordability.	4.82	3.35	2
5	Utilize an app or downloadable content (avoid reliance on high‐speed internet).	3.88	3.41	2
6	Avoid the need for elaborate technical tools.	4.12	3.82	4
11	Ensure transferability across borders by avoiding country‐specific technical specifications.	3.53	3.12	1
16	Offer free or affordable online cognitive‐behavioural therapy.	4.18	3.18	2
17	Address the digital divide and consider the absence of computers/internet at home for some.	4.41	3.24	2
19	If technology‐based, design for easy user adoption.	4.35	3.71	4
21	Keep it simple (minimize unnecessary technology if not required).	4.29	4.06	4
25	Respect people's modality preferences (in‐person, video, online tools, etc.).	4.53	3.65	4
30	Simplify communication for accessibility to both youth and adults.	4.82	4.65	4
31	Ensure accessibility for the hearing impaired, visually impaired and those with mobility constraints.	4.76	3.65	4
32	Make technology‐based solutions intuitive.	4.47	3.88	4
45	Realistically implement within the healthcare network (*Réseau de la santé et des services sociaux* in Quebec).	4.59	3.29	2
54	Ensure free and accessible availability to all people who stutter in Quebec, regardless of age and geographical origin.	4.88	2.76	2
94	Provide bilingual content (English/French) with easy translation into other languages.	4.24	3.82	4
95	Account for individual digital literacy levels and the reality of disadvantaged socioeconomic backgrounds.	4.59	3.65	4
111	Include remote training for clinicians and people who stutter (to save time and cost).	4.59	4.18	4
63	Utilize accessible technologies applicable in various settings (family, clinics, schools, healthcare system).	4.41	3.12	2
Cluster 4: Inclusive and Sustainable Intervention Design and Delivery		4.27	3.76	
35	Include training (or informational sessions) for people who stutter, provided by a community organization (minimizing costs as much as possible).	4.00	3.59	4
8	Offer training to as many clinicians as possible (e.g., across all regions of Quebec).	4.71	3.59	4
14	Intervention could be conducted by caregivers or healthcare professionals.	4.06	3.47	2
24	Ensure diversity in terms of gender, origin, etc. (if using videos or virtual worlds, for instance).	4.53	4.47	4
26	Have the intervention delivered by a speech therapist, teacher, psychologist, specialized educator.	4.18	3.82	4
53	Develop the intervention in Minimum Viable Product mode to create a tool based on user feedback, not creator assumptions.	4.24	3.53	2
65	Delivered by specialized clinicians in stuttering (speech therapists, social workers, psychologists, etc.).	4.47	4.06	4
73	Include people who stutter as mentors in the intervention.	3.88	3.59	4
79	Facilitated by at least one person who stutters.	3.76	3.88	3
89	Customizable and adapted to individual needs.	4.71	3.41	2
93	Engage people who stutter in creation and promotion.	4.59	4.12	4
98	Involve individuals with disabilities in tech tool development.	4.06	3.59	4
106	Include technology developers alongside people who stutter from the ideation phase.	4.47	4.00	4
108	Consider an implementation plan that ensures the sustainability of the innovation.	4.18	3.53	2
50	Reduce the carbon footprint of travel generated by the intervention (flexible location not too far from participants' residences).	4.24	3.00	2
75	Consider providing water and/or food in environmentally friendly containers (glass cups filled from an iron/ceramic carafe, re‐usable utensils, cloth napkins).	3.71	4.00	3
Cluster 5: Engaging and Interactive Multi‐Modal Approach		3.73	3.51	
12	Design an engaging experience that brings fun and relaxation. For instance, use multiple microphones to transform stutter sounds into captivating visuals projected within a room.	3.24	2.94	1
22	Ensure a minimum duration of 30 min and schedule sessions 1–3 times per week.	3.47	3.29	1
29	Deliver the theoretical content through online courses enriched with examples and videos. Children and adolescents benefit from repeated exposure to aid in assimilation.	3.65	3.82	3
41	Keep each session within a maximum duration of 15–20 min to enhance participants' focus and attention.	3.41	3.47	1
48	Incorporate art forms like painting, writing and drawing as expressive outlets for people who stutter.	3.59	3.76	3
49	Enhance visual appeal with engaging graphics and vibrant colours.	4.29	4.24	4
67	Introduce awareness‐raising books tailored for classrooms, people who stutter and their immediate circles.	4.18	3.82	4
78	Conduct virtual stigma workshops for the broader public.	4.00	3.47	2
80	Offer a hybrid approach that combines in‐person sessions led by speech therapists with individual interventions utilizing technological tools.	4.00	3.35	2
88	Suggest, for instance, a series of 15 1‐h cognitive‐behavioural therapy sessions, encompassing cognitive therapy, anxiety management, virtual reality exposure and role‐playing activities.	3.76	3.12	1
91	Implement a staged approach involving exposure to both virtual and real‐life scenarios.	4.00	3.65	4
96	Create a game‐like card set or interactive course structure catering to people who stutter.	3.35	3.35	1
101	Facilitate virtual reality–based group meetings for people who stutter, featuring avatars for enhanced interaction.	3.12	2.88	1
103	Demonstrate a balanced ‘dosage’ model, such as organizing five 1‐h sessions every 2 weeks, integrating theoretical content, practical exercises and group discussions.	4.18	4.00	4
Cluster 6: Flexibility		3.89	3.49	
18	Deliver group therapy in an online or in‐person format.	4.06	3.65	4
57	Provide the option for individual, autonomous and asynchronous use.	3.82	3.35	2
60	Tailor the program's duration and frequency to each individual's pace (similar to a purchased online course).	3.82	3.47	2
68	Adapt the intervention to various types of interventions offered.	4.06	3.24	2
84	Should be adaptable to suit various speaking rhythms.	3.71	3.76	3

### 3.2 Concepts Maps

**FIGURE 1:**
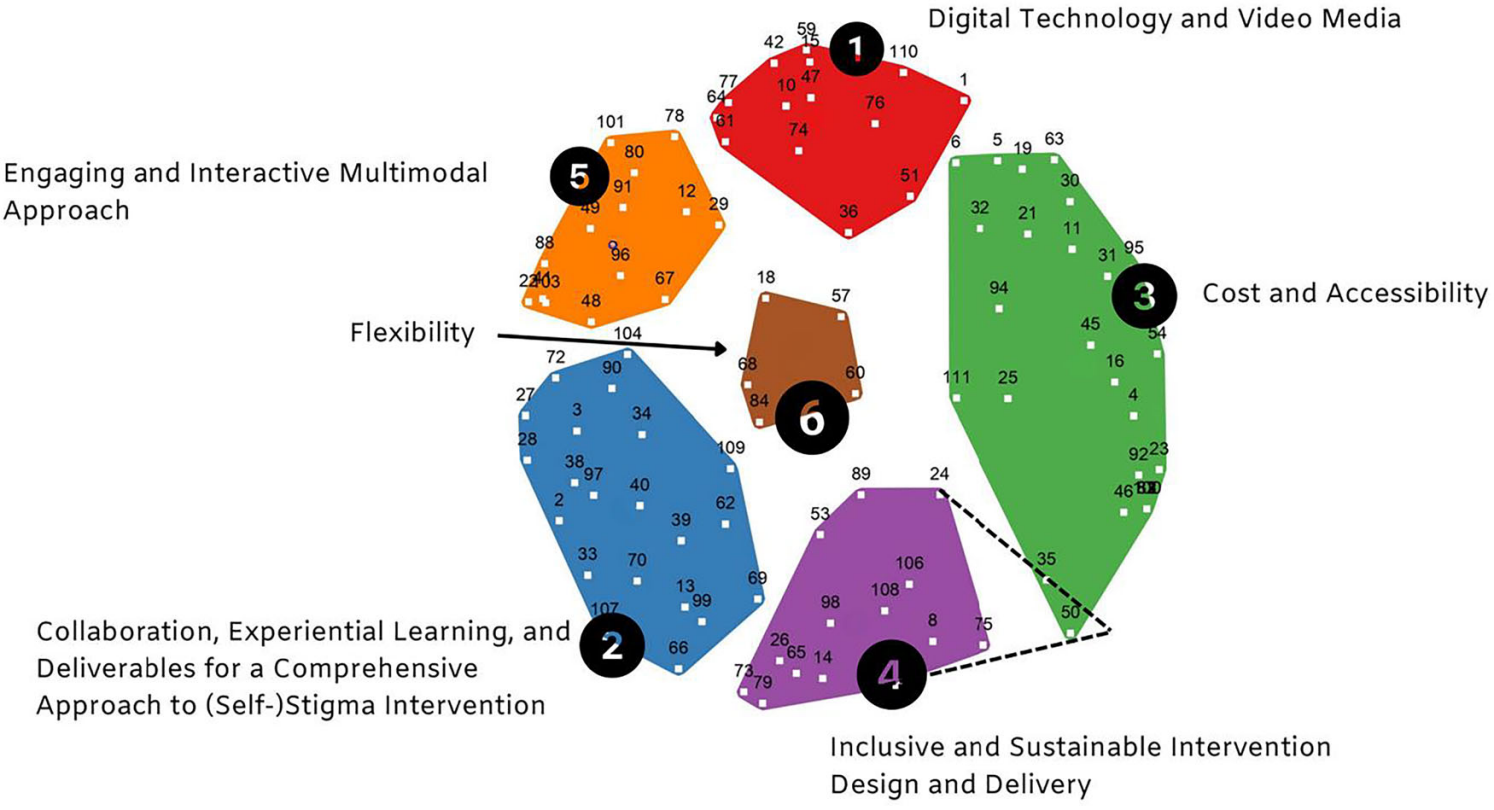
Cluster map.

**FIGURE 2:**
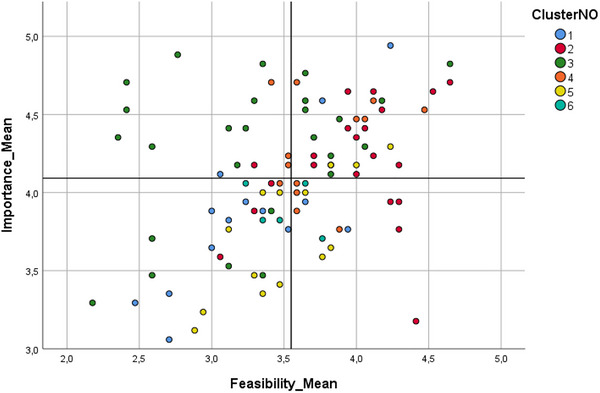
Go‐Zone graph.

The cluster map encompasses six clusters, with each containing between 5 and 26 ideas. During our interpretation and validation workshop, stakeholders recommended relocating ideas #35 and #50 to Cluster 4, believing this adjustment would improve thematic coherence. Figure 1 displays the Cluster Map, with dashed lines indicating these stakeholder‐driven re‐alignments. Additionally, Figure 2 presents a Go‐Zone graph for all 94 ideas, with the four quadrants demarcated by mean importance (4.09) and feasibility (3.55) ratings.

### 3.3 Feasibility and Importance of Ideas

Table 2 shows the distribution of ideas across the four quadrants. The top‐rated ideas for each cluster include: #36, ‘Ensure confidential data handling (secure platform)’ (feasibility mean 4.24, importance mean 4.94), #39, ‘Maintain a professional approach’ (feasibility mean 4.71, importance mean 4.65), #30, ‘Simplify communication for accessibility to both youth and adults’ (feasibility mean 4.82, importance mean 4.65), #24, ‘Ensure diversity in terms of gender, origin, etc.’ (feasibility mean 4.53, importance mean 4.47), #49, ‘Enhance visual appeal with engaging graphics and vibrant colors’ (feasibility mean 4.29, importance mean 4.24) and #18, ‘Deliver group therapy in an online or in‐person format’ (feasibility mean 4.06, importance mean 3.65).

**TABLE 2:** Proportion of ideas in each quadrant across the six clusters.

		Quadrant 1	Quadrant 2	Quadrant 3	Quadrant 4
Cluster Name	# Cluster	*N* (%)	*N* (%)	*N* (%)	*N* (%)
Digital Technology and Video Media	1	5 (35.7)	5 (35.7)	1 (7.1)	3 (21.4)
Collaboration, Experiential Learning and Deliverables for a Comprehensive Approach to (Self‐)stigma Intervention	2	1 (4.8)	3 (14.3)	2 (9.5)	15 (71.4)
Cost and Accessibility	3	5 (19.2)	11 (42.3)	N.D.	10 (38.5)
Inclusive and Sustainable Intervention Design and Delivery	4	N.D.	5 (31.3)	2 (12.5)	9 (56.3)
Engaging and Interactive Multi‐Modal Approach	5	6 (42.9)	2 (14.3)	2 (14.3)	4 (28.6)
Flexibility	6	0 (0.0)	3 (60.0)	1 (20.0)	1 (20.0)

*Note*: Quadrant 1 (low importance, low feasibility), Quadrant 2 (high importance, low feasibility), Quadrant 3 (low importance, high feasibility) and Quadrant 4 (high importance, high feasibility).

## 4 Discussion

This study aimed to explore stakeholders' perspectives on the RIH framework's values for reducing stigma‐based health inequalities among PWS. A total of 94 ideas were generated and organized into six thematic clusters. This discussion is structured around three key points, offering a guided reflection on stakeholders' engagement with the RIH framework.

### 4.1 Stakeholders’ Interaction With the Values of the RIH Framework

In analysing the stakeholder‐generated ideas through the RIH framework, our focus is not on judging the ideas themselves but on identifying which RIH values emerge more prominently in the data. Population health, for instance, is well‐represented through ideas like therapeutic groups (Cluster 2) that address mental health challenges faced by PWS, aligning directly with health needs and promoting inclusivity by reducing stigma. The health system value is also evident, particularly in the inclusiveness of the innovation process. Ideas involving PWS in the co‐creation of interventions (Clusters 2 and 4) reflect RIH's inclusiveness and responsiveness principles, offering scalable, sustainable solutions that can integrate into existing health systems. Economic value is emphasized through the focus on cost‐effective solutions, with several interventions proposed as low‐cost or free (Cluster 3) and anticipated to be supported by government and non‐profit funding. Organizational value appears in business models that prioritize sustainability and long‐term user benefits, exemplified in the development of an intervention kit (Cluster 2) and low‐maintenance technology (Cluster 1).

In contrast, environmental value is less prominent in the findings. Although only a few participants raised environmental considerations (e.g., using sustainable materials or reducing travel emissions), their relative absence suggests that this domain is not currently front‐of‐mind for most stakeholders. This limited focus on environmental sustainability aligns with the immediate priorities of clinicians, who often concentrate on addressing health inequalities at the individual level, with a more immediate focus on health outcomes (Lamoureux et al. 2024). For example, SLTs tend to work within frameworks that prioritize direct patient care and functional outcomes and are less accustomed to considering the broader societal or environmental implications of their interventions. Their training typically emphasizes improving individual health and social participation, with less focus on how these services intersect with sustainability goals or contribute to system‐level environmental impacts (Sherratt 2021). Moreover, there is a lack of standardized approaches to guide stakeholders in making informed decisions about reducing the environmental impact, such as carbon footprint, of health interventions (Lokmic‐Tomkins et al. 2022). As a result, we hypothesize that environmental sustainability remains somewhat peripheral in clinical practice—a tendency that can be observed across many health professions, but perhaps most acutely in rehabilitation, where the focus is often on measurable, client‐centered goals.

### 4.2 Gap Between the Perceived Importance and Feasibility of Health Innovations

The study reveals a notable gap between the perceived importance (mean 4.09) and feasibility (mean 3.55) of proposed ideas, emphasizing the challenge of translating impactful concepts into practical solutions. This observation aligns with recent research on the practical implementation of RRI principles, which identifies a similar tension between desirable attributes and their feasibility in real‐world settings (Rivard and Lehoux 2020). This discrepancy is particularly pronounced in high‐tech solutions such as virtual reality (VR). One reason explaining this may be that they have been identified in the literature as often requiring significant technical expertise, infrastructure and funding, with development and maintenance costs presenting additional obstacles within diverse healthcare settings (Kouijzer et al. 2023).

Interestingly, stakeholders could have rated these high‐tech solutions as important not for the technologies themselves, but for the therapeutic objectives they symbolize. This distinction raises an important question: Are high‐cost technologies essential to achieve specific therapeutic goals, or might alternative, low‐cost approaches be equally effective? The prominence of high‐tech solutions could also reflect a form of technological optimism, which, while promising, may inadvertently narrow perspectives on addressing complex issues like stigma. This optimism risks overshadowing simpler, community‐based interventions that might be more accessible and better aligned with a holistic view of care. Overreliance on digital tools could create a reductionist perspective, suggesting that these tools alone are sufficient to address complex public health challenges (Iyamu et al. 2022). Additionally, the accessibility of these technologies is not universal, potentially exacerbating health disparities, particularly among individuals with limited access to digital resources or from disadvantaged backgrounds (Yao et al. 2022). This issue underscores the importance of not allowing digital technological optimism to overshadow critical assessments of digital technologies’ impact on health equity and social justice (Alami et al. 2024; Gómez‐Ramírez et al. 2021).

### 4.3 Ensuring Long‐Term Sustainability for Health Innovations

Ensuring the sustainability of health innovations, especially digital health innovations, is crucial to preserving their impact over time (Côté‐Boileau et al. 2019). As technology advances rapidly, it is essential to design flexible, adaptable interventions that can evolve without prohibitive costs or disruptions to existing systems. Sustainability is shaped by infrastructure and digital literacy (Bhatia 2021), which can vary widely across populations, including among PWS (Wubineh et al. 2023). Achieving this requires regular updates that align with emerging technological advancements and best practices. Financially, sustainability demands efficient initial investments and strategies to manage ongoing costs for maintenance, updates and user support (Liaropoulos and Goranitis 2015), which may include diversified funding sources and targeted cost‐management strategies to enhance long‐term viability.

As noted in the previous section, digital health technologies may inadvertently widen health disparities if implemented without attention to access and usability barriers. To ensure that these innovations are inclusive and equitable for PWS, a balance between digital and non‐digital modalities can and should be considered. Although digital health technologies offer considerable benefits in terms of scalability and efficiency, they may inadvertently widen health disparities if implemented without attention to access and usability barriers. An overreliance on digital tools can neglect the needs of marginalized groups and risk undermining health equity efforts (Rodriguez and Lyles 2023; van de Vijver et al. 2023). Building on this concern, a hybrid approach combining digital platforms with community‐based or in‐person strategies has been recommended to enhance accessibility and ensure that interventions remain sensitive to the contexts and preferences of diverse populations (Kim and Backonja 2025). This is especially important in the context of stuttering, where face‐to‐face communication, social connection and contextual support—such as those provided through support groups and strong therapeutic alliances—can significantly influence therapeutic success and enhance self‐esteem (Boyle 2013; Connery et al. 2022).

Integrating new interventions into existing healthcare practices introduces its own challenges, making early and continuous collaboration with health services essential for seamless integration (Borges do Nascimento et al. 2023). Organizational readiness, including leadership support, a robust green strategy and adequate resources, has emerged as a critical factor for success (Alami et al. 2023). Regular evaluations of health innovations, which track both short‐ and long‐term health impacts, are vital for ensuring continued relevance and adaptability within the healthcare landscape. To support meaningful implementation in the context of stuttering, these evaluations should also consider outcomes beyond fluency, including identity, participation and communicative confidence. While the importance of such innovations is recognized, their practical feasibility remains a key concern for widespread adoption.

### 4.4 Lessons Learned and Moving Forward

A key lesson from our findings is that sustainability remains a peripheral concern for many stakeholders in rehabilitation, particularly among clinicians, as reflected in the limited references to environmental value across clusters. Clinicians may not always perceive the connection between their everyday decisions and broader issues of resource conservation, ecological impact or the long‐term sustainability of patient care (Alami et al. 2023). Embedding sustainability more explicitly into clinical education and ongoing professional development could help practitioners recognize and act on their capacity to influence systemic health outcomes over time. Such initiatives could help shift sustainability from a peripheral consideration to a more integrated component of clinical decision‐making, aligning patient care not only with immediate health outcomes but also with ecological and social responsibility. Moving forward, integrating sustainability checkpoints within clinical guidelines and fostering collaboration with HIs are promising strategies for cultivating what might be called a ‘sustainability reflex’ within the healthcare field.

Another key insight from this study is the critical role of incorporating patient and user feedback to bridge the gap between impactful concepts and practical, implementable solutions. Engaging stakeholders across the lifecycle of innovation—including design, implementation and evaluation—is essential for developing balanced approaches between digital and non‐digital interventions. By actively integrating feedback from patients, clinicians and policymakers, researchers can create frameworks that prioritize not only sustainability but also equity and real‐world applicability (Côté‐Boileau et al. 2019). This is particularly important in the context of stuttering, where interventions must respond to the unique communicative, emotional and identity‐related experiences of PWS (Sønsterud et al. 2020). Integrating feedback from PWS can help ensure that interventions promote not only fluency or communicative efficiency, but also self‐acceptance, agency and meaningful participation in everyday life. This inclusive, responsive approach supports the translation of innovative ideas into sustainable practices, carefully evaluating the added value of digital innovations and avoiding an over‐reliance on technology that might not suit all users. In doing so, we can ensure that health innovations remain grounded in the diverse needs of those they aim to serve, achieving outcomes that are both meaningful and enduring.

### 4.5 Limitations of the Study

The findings of this study should be interpreted with some limitations in mind. First, while generalizability was not a primary objective, our results reflect the perspectives of a specific group of stakeholders with unique experiences and priorities. These perspectives may not fully capture the broader views of all PWS, clinicians or HIs. The sample size is relatively small, and the findings are rooted in the sociocultural and institutional context of Québec, Canada, a region with its own healthcare structures, cultural norms and language landscape. These contextual factors likely influenced participants' understanding of stigma, feasibility and systemic barriers. Future research should aim to expand the sample to include participants from other provinces or countries, particularly in regions with different healthcare systems, cultural values or linguistic communities. Such studies would help assess the transferability of the findings and provide a more comprehensive picture of how responsible health innovation principles are interpreted across diverse contexts. Participatory concept mapping has successfully been conducted with larger and more heterogeneous groups, showing that the method is scalable and suitable for multi‐sectoral engagement (Johnson et al. 2020). Second, some selection bias may have occurred in the recruitment process. Participants were identified through targeted outreach within networks likely to be sympathetic to equity‐oriented and participatory approaches. This may have led to overrepresentation of individuals already invested in inclusive or innovation‐driven perspectives. The subjective ratings provided by stakeholders reflect their distinct professional and personal backgrounds, which influence their assessments of importance and feasibility. This focus could limit the broader applicability of our findings, highlighting the need for future studies to incorporate a more diverse range of stakeholder perspectives to explore how varied experiences shape evaluations of health innovations. Third, there was a limited emphasis on environmental value in our findings, which we interpret not as an incidental gap but as a potential indicator of broader educational needs within the field. The 1‐h training prior to data generation may not have been sufficient for stakeholders to grasp the full implications of sustainability within health innovations. This finding suggests a need for more detailed training or workshops that give explicit focus to sustainability and its practical applications in health innovation. Replicating the study with an extended workshop that places greater emphasis on sustainability could offer valuable insights into stakeholders’ responses to these critical aspects.

## 5 Conclusion

This study demonstrates how the RIH framework can support the design of stigma‐reducing health innovations that are grounded in the lived experiences of PWS. The stakeholder‐generated ideas reflect a clear desire for interventions that promote systemic change, extending beyond individual‐level remediation toward collective health justice for PWS. In doing so, this study positions stuttering not simply as a communication issue, but as a multi‐faceted condition entangled with stigma‐based health inequalities. By applying RIH, this study highlights how responsible innovation can serve as a strategic tool for advancing sustainability, inclusion and equity in SLP practices, ensuring that stakeholder voices guide the entire innovation process and that technological potential is aligned with the real‐world needs of PWS. In centering equity and lived experience, RIH moves us closer to interventions that actively work to transform the stigma faced by PWS.

## Conflicts of Interest

The authors declare no conflicts of interest.

## Data Availability

The data that support the findings of this study are available from the corresponding author upon reasonable request.
